# Oriented Cell Alignment Induced by a Nanostructured Titanium Surface Enhances Expression of Cell Differentiation Markers

**DOI:** 10.3390/nano9121661

**Published:** 2019-11-22

**Authors:** Maria Antonia Llopis-Grimalt, Andreu Miquel Amengual-Tugores, Marta Monjo, Joana Maria Ramis

**Affiliations:** 1 Group of Cell Therapy and Tissue Engineering, Research Institute on Health Sciences (IUNICS), University of Balearic Islands, Ctra Valldemossa km 7.5, 07122 Palma, Spain; mantonia.llopis@uib.es (M.A.L.-G.); andreu.amengual.aat@gmail.com (A.M.A.-T.); 2 Balearic Islands Health Research Institute (IdISBa), 07120 Palma, Spain

**Keywords:** nanostructuration, mechanotransduction, nanonets, nanopores, implant–tissue integration

## Abstract

A key factor for dental implant success is a good sealing between the implant surface and both soft (gum) and hard (bone) tissues. Surface nanotopography can modulate cell response through mechanotransduction. The main objective of this research was the development of nanostructured titanium (Ti) surfaces that promote both soft and hard tissue integration with potential application in dental implants. Nanostructured Ti surfaces were developed by electrochemical anodization—nanopores (NPs) and nanonets (NNs)—and characterized by atomic force microscopy, scanning electronic microscopy, and contact angle analysis. In addition, nanoparticle release and apoptosis activation were analyzed on cell culture. NP surfaces showed nanoparticle release, which increased in vitro cell apoptosis. Primary human gingival fibroblasts (hGFs) and human bone marrow mesenchymal stem cells (hBM-MSCs) were used to test cell adhesion, cytotoxicity, metabolic activity, and differentiation markers. Finally, cell orientation on the different surfaces was analyzed using a phalloidin staining. NN surfaces induced an oriented alignment of both cell types, leading in turn to an improved expression of differentiation markers. Our results suggest that NN structuration of Ti surfaces has great potential to be used for dental implant abutments to improve both soft and hard tissue integration.

## 1. Introduction

In dental implants, a good sealing between both soft (gum) and hard (bone) tissues and the implant surface is a key factor for the implant success and drastically reduces the risk of periimplantitis and the risk of the implant failure [1,2].

Surface topography can modulate cell behavior by a process called mechanotransduction without the need to change the surface chemical composition. The mechanical signals induced by the different surface topography features, such as pore diameter or pore interspace, are converted to biochemical signals, influencing the cell response to the surface [3,4,5,6].

Nanopatterned surfaces can induce a better organized cytoskeleton and a spread cell morphology, which is known to be beneficial for osteogenic differentiation of mesenchymal stem cells (MSCs) [4,6]. In addition, this capacity to modulate cell behavior through topographical features has been observed using different cell types and could be a strategy to modulate both soft and hard tissue integration.

A nanoscale geometry can be achieved on Ti surfaces using different approaches; with electrochemical anodization being one of the most frequently used [7]. Depending on the electrolyte composition and the reaction conditions, different nanostructures and morphologies can be achieved by electrochemical anodization [5,7,8]. These nanoscale morphology modifications have been proven to have an important effect on cell behavior. Small pore diameters (less than 100 nm) have been shown to promote cell adhesion, proliferation, and differentiation, while bigger diameters can promote cell apoptosis [6,9,10].

Although a lot of research is being developed to improve the bone tissue integration of Ti implants, few studies assess both soft and hard tissue integration at the same time. In this study, we aimed to produce and characterize different nanostructured Ti surfaces that promote both human gingival fibroblasts’ (hGF, as a soft tissue model) and human bone-marrow mesenchymal stem cells’ (hBM-MSC, as a hard tissue model) adhesion and differentiation. Two different nanostructures on TiO_2_ were produced by changing anodization parameters and characterized by atomic force microscopy (AFM), scanning electron microscopy (SEM), and contact angle analysis. Then, we analyzed nanoparticle release from the surface and its effect on hGFs. Finally, we evaluated the adhesion and differentiation of the different surfaces on hGFs and on hBM-MSC.

## 2. Materials and Methods

### 2.1. Materials

Machined titanium discs—c.p. grade IV, 6.2 mm in diameter, and 2 mm in height—were purchased from Implantmedia (Lloseta, Spain).

### 2.2. Surface Nanostructuration

Ti discs were polished and cleaned as previously described [11]. Afterwards, two nanostructures were produced (nanonets, NNs; and nanopores, NPs) using different anodization conditions using an Autolab (Metrohm Autolab BV, Utrecht, The Netherlands), with the Ti samples as anode and a platinum electrode (Metrohm Autolab BV, Utrecht, The Netherlands) as cathode. For the production of NN, polished titanium discs were anodized in an ethylene glycol based electrolyte (0.1 M NH_4_F, 8 M H_2_O) with a first anodization of 30 min at 35 V and a second one of 10 min at the same voltage. For the production of NP, polished titanium discs were anodized in an ethylene glycol-based electrolyte (0.1 M NH_4_F, 1 M H_2_O) with a first anodization of 30 min at 60 V and a second one of 10 min at the same voltage. In both protocols, a peeling was done between the first and second anodization using Scotch^®^ MagicTM tape (3M, Maplewood, MN, USA).

### 2.3. Surface Characterization

The morphology of the different surfaces was analyzed using scanning electron microscopy (SEM). Samples were sputter gold coated before SEM analysis. Images were acquired using a scanning electron microscope (SEM; Hitachi S-3400 N, Krefeld, Germany) using secondary electrons, vacuum conditions, and 15 kV of voltage. Images were analyzed using ImageJ software (National Institutes of Health, Bethesda, MD, USA) to determine pore diameter.

The topography of the samples was analyzed using an atomic force microscope (VECCO model multicode, VECCO, Plainview, Oyster Bay, NY, USA) in air tapping mode with a scan size of 10 µm in combination with HQ:NSC35/Al probes (Mikromasch, Lady’s Island, SC, USA) with a nominal spring constant of 16 N/m and resonance frequency of 300 kHz.

The static contact angle was calculated by the sessile drop method using a Nikon D3300 (AF-P DX 18–55 mm lent). The contact angle measurements were performed using four samples of each group using 2 µL ultrapure water as a wetting agent. Image analyses were performed using ImageJ software (National Institutes of Health, Bethesda, MD, USA).

### 2.4. Cell Culture

Two different donors of primary human gingival fibroblasts (hGF; Provitro GmbH, Berlin, Germany) were used (male:female ratio 1:1). Provitro assured that cells were ethically and legally obtained and all donors provided written informed consent. Cells were cultured at 37 °C/5% CO_2_, and maintained in low glucose Dulbecco modified Eagles minimal essential medium (DMEM) GlutaMAX medium (Gibco, Life Technologies, Carlsbad, California, CA, USA) supplemented with 10% fetal calf serum (Biowest, Nuaillé, France), penicillin (100 µg/mL), and streptomycin (100 µg/mL) (Biowest, Nuaillé, France). Cells were seeded in 96-well plates at a density of 7.0 × 10^3^ cells per well and media were supplemented with ascorbic acid to favor collagen deposition (100 µM; Sigma-Aldrich, St. Louis, MO, USA). Cells were characterized in a previous study in our research group [12].

One donor of human bone marrow-mesenchymal stem cells (hBM-MSCs; Stemcell Technologies, Grenoble, France) was used (20 years, female). Stemcell Technologies assured that cells were obtained ethically and legally and that donors provided written informed consent. Cells were cultured at 37 °C/5% CO_2_, and maintained in low glucose DMEM GlutaMAX (Life Technologies, Carlsbad, CA, USA) supplemented with 10% stem cell-tested fetal bovine serum (Biosera, Boussens, France), penicillin (100 µg/mL), and streptomycin (100 µg/mL).

Cells were seeded in 96-well plates at a density of 7 × 10^3^ cells per well and grown for seven days prior to media supplementation with hydrocortisone 0.1 µM and ascorbic acid 50 µg/mL.

### 2.5. Analysis of Particle Release from the Different Surfaces and Its Effect on Biocompatibility

On one hand, the different Ti discs were incubated at 37 °C with DMEM for seven days in order to obtain conditioned media. On the other hand, hGFs (7 × 10^3^ cells per well) were seeded in a 96–well plate and allowed to grow until they achieved confluence. Then, conditioned media from Ti discs were added to the cells. After 48 h lactate dehydrogenase (LDH) activity was determined from culture media following the manufacturer’s instructions (Cytotoxicity Detection kit, Roche Diagnostics, Manheim, Germany) and total metabolic activity was analyzed using Presto Blue reagent following the manufacturer’s protocol, at 1 h of reagent incubation time. Apoptosis activation was analyzed using caspases 3/7 activation with the Caspase-Glo^®^ 3/7 assay (Promega, Madison, WI, USA). Finally, another set of Ti discs was incubated at 37 °C with ultrapure water for seven days in order to measure nanoparticle release by dynamic light scattering (DLS) using a Zetasizer ZS90 (Malvern Panalytical Ltd., Malvern, UK) and a ZetaView^®^ nanoparticle tracking analyzer (NTA) (ParticleMetrix GmbH, Meerbusc, Germany).

### 2.6. Bioactivity of Nanostructured Surfaces

The bioactivity on hGFs and hBM-MSCs was only assessed using NN surfaces.

### 2.7. Cell Adhesion

hGFs and hBM-MSCs were allowed to adhere for 30 min to the different surfaces. Unbounded cells were removed by washing twice with PBS and cell adhesion was analyzed using Presto Blue reagent (Life Technologies, Carlsbad, CA, USA) following the manufacturer’s protocol, at 24 h of reagent incubation time.

### 2.8. Cytotoxicity Assay

After 48 h of culture, LDH activity was determined from culture media of both cell types following the manufacturer’s instructions (Cytotoxicity Detection kit, Roche Diagnostics, Manheim, Germany). The results were presented relative to the LDH activity in the medium of cells cultured in tissue culture plastic (low control, 0% of cell death) and of cells growing on tissue culture plastic treated with surfactant Triton X-100 1% (high control, 100% of cell death).

### 2.9. Metabolic Activity

Total metabolic activity was analyzed at 48 h, 7 d, and 14 d for hGFs and at 48 h, 8 d, and 15 d for hBM-MSCs culture using Presto Blue reagent (Life Technologies, Carlsbad, CA, USA), following the manufacturer’s protocol, at 1 h of reagent incubation time.

### 2.10. Collagen Quantification

After 14 d of culture, hGFs were washed with phosphate buffer saline (PBS) (Biowest, Nuaillé, France), dried overnight at 37 °C in a humidified atmosphere and dried for 24 h at 37 °C in a dry atmosphere. Collagen was stained with 0.1% Sirius red F3BA (Sigma, Saint Louis, Missouri, MO, USA) in saturated picric acid (Sigma, Saint Louis, Missouri, MO, USA) for 1 h. Unbound die was removed by washing with 10 mM HCl (Scharlab, Barcelona, Spain), and dye was solubilized with 100 mM NaOH (Scharlab, Barcelona, Spain). Absorbance was measured with a microplate reader at 540 nm.

### 2.11. Alkaline Phosphatase (ALP) Activity

After 15 d of culture, ALP activity of hBM-MSC lysate was analyzed as previously described [13].

### 2.12. Phalloidin-Fluorescein Isothiocyanate (FITC)

Cells were fixed in paraformaldehyde (4%, 15 min) and permeabilized with Triton X-100 (0.25%, 10 min). After that, cells were stained with Phalloidin-FITC (5µg/mL, 30 min, Sigma-Aldrich) and mounted with 4′,6-diamidino-2-fenylindol (DAPI) Fluoroshield (Sigma-Aldrich). Samples were visualized using a confocal microscope (Leica DMI 4000B equipped with Leica TCS SPE laser system, Wetzlar, Germany) and image analysis was performed with ImageJ software (National Institutes of Health, Bethesda, MD, USA).

### 2.13. Statistical Analysis

All data are presented as mean value ± standard error of the mean (SEM). The Kolmogorov–Smirnov test was done to assume parametric or non-parametric distributions. Differences between groups were assessed by one-way analysis of variance (ANOVA) test using Bonferroni as post hoc or by Kruskall Wallis depending on their normal distribution when more than two experimental groups were compared; when two groups were compared, the differences between groups were assessed by Student’s *t*-test. SPSS software (version 25.0, Chicago, IL, USA) and GraphPad Prism (version 7, La Jolla, CA, USA) were used. The results were considered statistically significant at *p*-values < 0.05.

## 3. Results

### 3.1. Characterization of Surface Topography and Wettability

**Figure 1:**
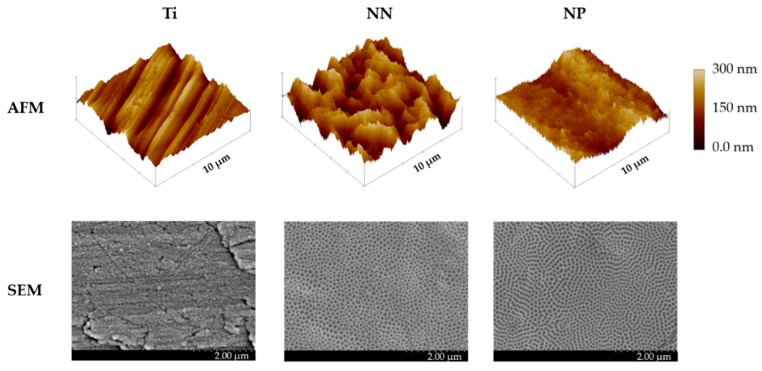
Physical characterization of nanostructured surfaces. Representative AFM (atomic force microscopy) and SEM (scanning electron microscopy) images of the different nanostructured titanium surfaces. Scale bars for AFM images represent 10 µm and those for SEM images represent 2.0 µm. NP, nanopore; NN, nanonet.

Two different nanostructures were obtained with the anodization conditions used as demonstrated by the SEM images: NP and NN (Figure 1). On one hand, higher voltage and low H_2_O content produced an NP structure on the surfaces; on the other hand, by decreasing voltage and increasing H_2_O content, an NN structure was produced (Table 1).

The nanostructures obtained showed different surface roughness and wettability (Table 1). Thus, the average surface roughness (Sa) of the obtained NN surfaces was higher than NP and Ti. Meanwhile, water contact angle (CA) measurements indicated that, although all tested surfaces are hydrophilic (CA lower than 90°), NP surfaces are more hydrophilic than those of NN and Ti.

### 3.2. Analysis of Particle Release from the Different Surfaces and Its Effect on Biocompatibility

**Figure 2:**
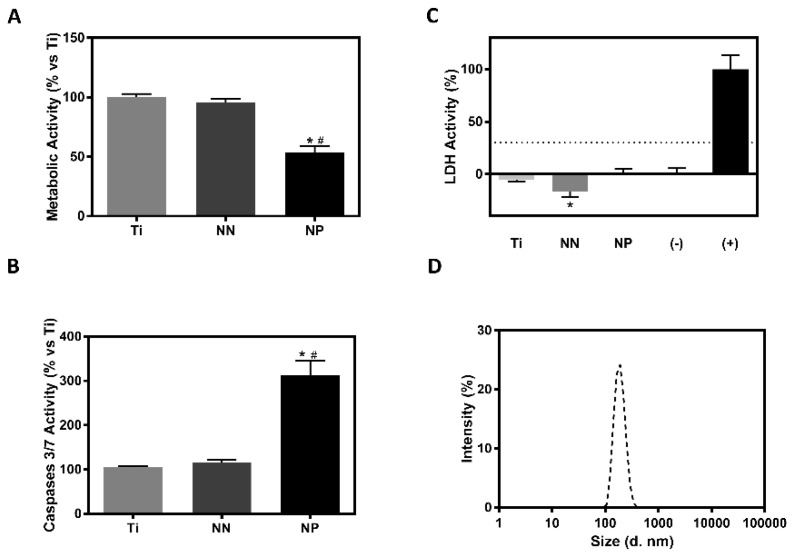
Analysis of particle release from the different surfaces and its effect on biocompatibility. Values represent the mean ± SEM (*n* = 6; A, B, C) (*n* = 3; D). (**A**) Metabolic activity (% vs. Ti) of human gingival fibroblasts (hGFs) cultured with conditioned media. (**B**) Caspases 3/7 Activity (% vs. Ti) of hGFs cultured with conditioned media. (**C**) LDH activity (% vs. (+)) of hGFs cultured with conditioned media; hGFs cells cultured with tissue culture plastic (TCP) conditioned media are considered (-) and hGFs cells treated with Triton X-100 1% are considered (+). Results were statistically compared by analysis of variance (ANOVA) and Bonferroni as post hoc: * *p* < 0.05 versus Ti for LDH activity and by Kruskall Wallis for metabolic activity and caspases 3/7 activity: * *p* < 0.05 versus Ti; # *p* < 0.05 versus NN. (**D**) Diagram that represents the size distribution and the intensity of the nanoparticles in water cultured with NP surfaces, analyzed with a Zetasizer.

The first step that we undertook in order to assess the biocompatibility of the obtained surfaces was to analyze cytotoxicity and metabolic activity of hGF cells using media conditioned with the modified surfaces to test whether there was indirect toxicity on the cells. hGF cells cultured with NP conditioned media showed lower metabolic activity compared with the other groups, although no significant differences on cytotoxicity levels were found (Figure 2). In addition, the NP group showed higher caspases 3/7 activity (Figure 2).

The presence of nanoparticles released from the surfaces was evaluated through DLS and NTA. Only NP surfaces showed nanoparticle release (Figure 2), obtaining a peak between 100 nm and 1000 nm. In addition, these particles presented a mean size of 220.5 nm and a concentration of 1.08 × 10^9^ particles/mL. Taking all these results together, NP surfaces were excluded from further studies.

### 3.3. Bioactivity of NN Surfaces

In order to determine the potential effects of nanostructuration on gum tissues and its osteogenic capacity, the biological activity of the NN surface was evaluated with hGFs and hBM-MSCs.

**Figure 3:**
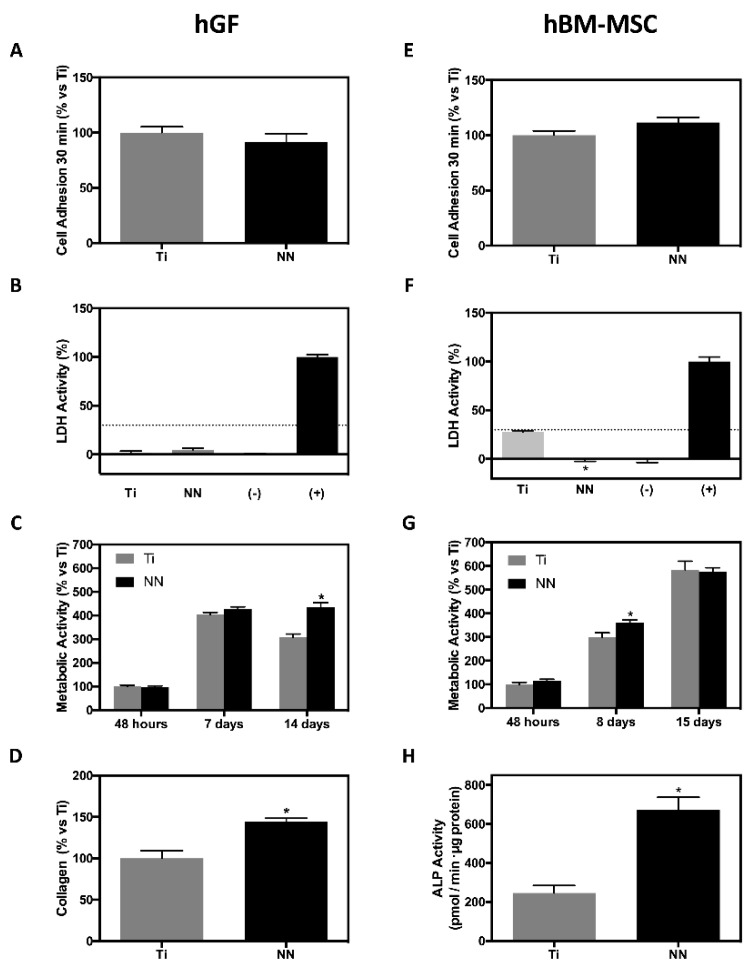
Surface bioactivity on hGFs and human bone marrow mesenchymal stem cells (hBM-MSCs). The graphs on the left (**A**–**D**) represent hGFs’ response to NN surface (*n* ≥ 9) and the graphs on the right (**E**–**H**) represent hBM-MSCs’ response to NN surfaces (n ≥ 6). (**A**,**E**) Cell adhesion to the surfaces, expressed as % versus Ti. (**B**,**F**) Cytotoxicity of cells cultured on the different surfaces, measured as LDH activity; cells cultured on TCP are considered (-) and hGFs/hBM-MSCs treated with Triton X-100 1% are considered (+). Results are expressed as % versus (+). (**C**,**G**) Metabolic activity of cells cultured on the different surfaces over the time; results are expressed as % versus Ti. (**D**) Collagen deposition of hGFs cells cultured on the different surfaces for 14 days; results are expressed as % versus Ti. (**H**) ALP activity of hBM-MSCs cultured on the different surfaces for 15 days; results are expressed as % versus Ti. Results were statistically compared by Student’s *t*-test: * *p* < 0.05 versus Ti.

The adhesion of hBM-MSCs and hGFs to Ti and NN surfaces was tested 30 min after seeding (Figure 3), with both cell types showing similar adhesion to both NN and Ti surfaces. After 48 h of culture, no cytotoxicity was found (Figure 3), showing no significant differences between cells cultured onto NN surfaces (hGF) or significantly lower LDH activity levels (hBM-MSC) than the negative control.

Furthermore, we evaluated the effect of NN surfaces on hGF and hBM-MSC cells on metabolic activity at 48 h, 7 d, and 14 d of culture. Metabolic activity of hGFs was higher after 14 days of culture on NN surfaces compared with Ti, while hBM-MSC cultured on this nanostructured surface showed higher metabolic activity compared to Ti after 8 days of culture, although differences were lost after 15 days of culture (Figure 3).

In order to evaluate the effect of the obtained NN surface on hGFs and hBM-MSCs, collagen deposition (hGFs) and ALP activity (hBM-MSCs) were analyzed as functional markers. Collagen deposition of hGF cells after 14 days of culture was higher on NN compared with Ti. hBM-MSCs cultured on NN surfaces showed higher ALP activity compared with Ti (Figure 3).

**Figure 4:**
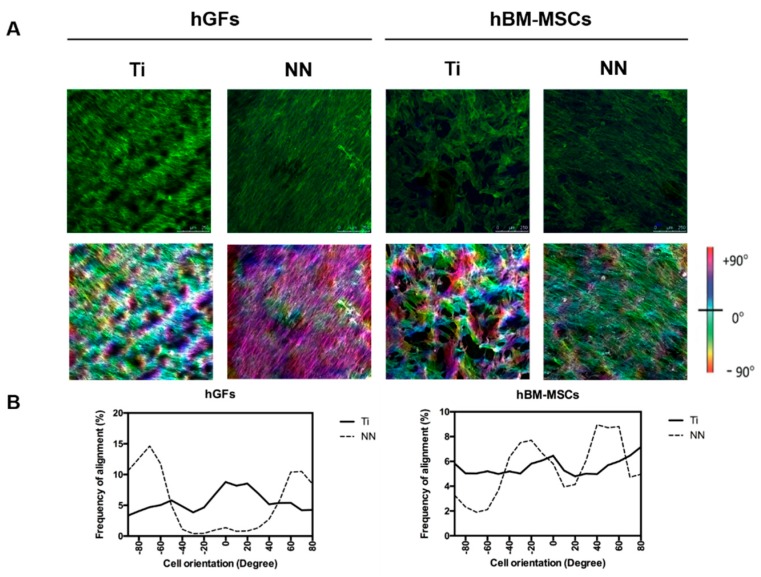
Cell orientation on the nanostructured surfaces. (**A**) The images in the upper row represent hGFs and hBM-MSCs stained with Phalloidin-FITC (green) and DAPI (blue) (*n* = 2). Images in the middle row show actin fibers orientation degree with different colours through the surface analysed using ImageJ software. (**B**) The graphs represent the % of pixels in each orientation angle for both cell types for each surface.

Finally, we analyzed the alignment and distribution of both cell types on Ti and NN surfaces. Image analyses showed that hGFs and hBM-MSC cultured on NN structured Ti surfaces exhibit a high frequency of alignment (Figure 4). In contrast, cells cultured on control Ti surfaces showed a random orientation, although being distributed through the entire surface area (Figure 4).

## 4. Discussion

Here, we have developed a nanostructured surface (NN surface) that induces an oriented alignment of both mesenchymal stem cells—as a bone model—and gingival fibroblasts—as a gum model. Moreover, both cells show an improved expression of the evaluated differentiation markers (ALP activity and collagen deposition).

The aim of our study was to find a nanostructured titanium surface that could improve both soft and hard tissue integration with potential application in dental implants, in order to have one unique surface for both the implant fixture and abutment. The surface characteristics of the implant play a key role in its interaction with the surrounding tissues. Increased roughness at the micro level promotes osseointegration of screw implants, but in implant abutments, there is a possibility of increased periimplantitis risk. In contrast, with certain modifications at the nano level, both soft and hard tissue integration are promoted [8,14].

Two different nanostructures were obtained with the anodization conditions used by changing the voltage and water content of the electrolyte: NP and NN. Although the obtaining of different morphologies by changing reactions parameters has been extensively reported by others [7,8,15], and there is prior research on NP surfaces [3,5], to the best of our knowledge, no previous reports have evaluated NN structures. It was not our intention to study such nanostructures, but, when setting up the surface modification, SEM images of the NN recalled us to the structure of trabecular bone (though at a different scale), so we decided to characterize these structures and to study their biocompatibility.

The biocompatibility of nanostructured Ti surfaces usually depends on the nanostructure porous size and composition. Because the nanostructure dimensions obtained for both surfaces are lower than 100 nm—which has been proven to enhance cell apoptosis [10]—the first step that we undertook in order to assess the biocompatibility of the surfaces was to analyze indirect cytotoxicity and metabolic activity of hGF cells using conditioned media with modified surfaces. Although no significant differences on cytotoxicity levels were found, hGF cells cultured with NP conditioned media showed lower metabolic activity compared with the other groups. These lower metabolic activity levels could be explained by the increased caspases 3/7 activity found, indicating cell apoptosis. This deleterious effect could be explained by the high concentration of released nanoparticles found in the NP group, which is in line with the toxic effect found for TiO_2_ nanoparticles on normal and cancerous epithelial oral cells [16,17]. In a previous study, it was found that nanoporous Ti foils also released nanoparticles, although no cytotoxic effect was found. However, the nanoparticle concentration in that study (2.85 × 10^8^ particles/mL) was lower compared with the concentration found in this study (1.08 × 10^9^ particles/mL) [5]. Therefore, NP surfaces were excluded from further studies.

In order to determine the potential effects of the NN structure on gum tissues and its osteogenic capacity, the biological activity of the surfaces was evaluated with hGFs and hBM-MSCs. On one hand, hGFs are the preferred cells to attach to the abutment of the implant after its installation [18]; these cells regulate collagen and proteoglycan metabolism, being responsible for the constant adaptation, wound healing, and regeneration of gingival connective tissue [19,20], thus constituting a valuable model for screening new implant abutment surfaces [12]. On the other hand, mesenchymal cells (MSCs) are recruited to form osteoprogenitor cells and, with time, develop into differentiated bone cells, being of utmost importance for proper bone healing or anchorage of an implant [21]. For this study, hBM-MSCs were selected owing to their capacity to regenerate alveolar bone, cementum, and periodontal ligament [22].

In agreement with previous reports [9], both cell types—hBM-MSCs and hGFs—showed similar adhesion to both NN and Ti surfaces. No cytotoxicity was found after 48 h of culture of the cells onto the surfaces, with cells cultured onto NN surfaces showing no significant differences (hGF) or significantly lower LDH activity levels (hBM-MSC) compared with the negative control. Other studies have demonstrated that hGFs’ proliferation after 14 days of incubation is higher on nanostructured surfaces compared with Ti, but no differences were found on the same study at lower incubation times [23], in agreement with our results.

Surface morphology and pore diameter are key factors for the surface in vitro effect. Small diameters induce cell differentiation, while bigger diameters can induce cell death and apoptosis [9]. Huang et al. reported that TiO_2_ nanotubes with a 30 nm diameter promote cell proliferation, while tubes with a 90 nm diameter decrease cell viability [24]. Other studies suggest that 15–20 nm is the optimal diameter size to promote cell adhesion, proliferation, and differentiation in nanotubular surfaces as it allows the formation of focal adhesion complexes [9,10]. Our NN surface presents a porous size of 77.7 ± 0.7 × 47.4 ± 0.5, which is bigger than the optimal size reported for nanotubular surfaces, but the biological properties shown could be related to the different surface morphology observed by SEM and AFM compared with the NP surface. On the other hand, other works show that bigger diameters (around 70 nm) do not impair cell spreading and differentiation, and that other parameters such as nanopore interspace influence cell response [6,25]. As a consequence of its topography, the NN surface showed higher roughness than the control Ti, which could benefit both soft and hard tissue integration, as the roughness values are in the nanolevel scale [9,26].

The connective tissue around a dental implant is characterized by collagen fibers aligned parallel to the implant abutment. Collagen deposition and orientation is fundamental for a good sealing and to prevent biofilm formation that could lead to periimplantitis [23,27]. Collagen deposition of hGF cells after 14 days of culture was higher on NN compared with Ti, as in other studies, which show an increased relative mRNA expression of Collagen-I on cells cultured for 14 days on nanostructured surfaces [23]. This effect could be very important as this higher collagen deposition could lead to a better soft tissue sealing around the implant abutment, preventing the onset of periimplantitis.

ALP activity is an early marker of osteoblast differentiation, which is involved in hydroxyapatite crystal deposition. hBM-MSCs cultured on NN surfaces showed higher ALP activity compared with Ti, indicating a higher differentiation towards the osteoblastic lineage, in agreement with other studies that have shown that nanotubular surfaces with a small pore diameter promote MSC differentiation to the osteoblastic lineage [9,28].

Finally, hGFs and hBM-MSCs cultured on NN surfaces showed a high frequency of alignment, in contrast to cells cultured on control Ti surfaces, which showed a random orientation. The increased cell differentiation induced by the NN surfaces observed in both cell types could be explained by this higher frequency of alignment. Previous studies have demonstrated that nanostructured surfaces can induce hBM-MSCs’ osseodifferentation [29]. In addition, it is known that surface topographical features can modulate cell response (adhesion, migration, proliferation, and differentiation) through mechanotransduction [3,4,30]. Specifically, MSCs’ differentiation is improved with a well-organized cytoskeleton and a well-spread cell morphology—features that we observe in cells cultured onto NN surfaces. In our study, we found a lack of effect on cell adhesion by the presence of NN on the surface, but this nanostructuration induced an oriented cell alignment for hGFs and hBM-MSCs, leading to an increased functionality (collagen deposition and ALP activity), indicating that this structure has a positive effect on cell differentiation.

## 5. Conclusions

In conclusion, we developed a NN nanostructured Ti surface that induces an oriented alignment of gingival fibroblasts and mesenchymal stem cells, leading in turn to an improved expression of differentiation markers. This nanostructured surface could have a potential application in dental implants for an improved soft and hard tissue integration.

## Figures and Tables

**Table 1:** Physical characterization of the different titanium surfaces. Values represent the mean ± SEM (*n* = 5). Ra = average roughness; Ssk = surface skewness; Sku = surface kurtosis. Results were statistically compared by analysis of variance (ANOVA) and Student’s *t*-test as post hoc: * *p* < 0.05 versus Ti; # *p* < 0.05 versus nanonet (NN) for Ra and Ssk and by Kruskall Wallis for Sku. NP, nanopore.

Parameter	Ti	NN	NP
Porous size (nm)	-	77.7 ± 0.7 × 47.4 ± 0.5	52.9 ± 0.9
Contact Angle (°)	71.7 ± 8.7	84.3 ± 3.8	17.7 ± 1.3
Ra (nm)	28.9 ± 0.7	55.8 ± 1.6 *	31.3 ± 1.9 #
Sku	6.78 ± 2.96	2.81 ± 0.13	3.74 ± 0.39
Ssk	0.34 ± 0.24	0.07 ± 0.04	0.20 ± 0.07
